# Higher levels of myelin phospholipids in brains of neuronal α-Synuclein transgenic mice precede myelin loss

**DOI:** 10.1186/s40478-017-0439-3

**Published:** 2017-05-08

**Authors:** Jessica Grigoletto, Katharina Pukaß, Ayelet Gamliel, Dana Davidi, Rachel Katz-Brull, Christiane Richter-Landsberg, Ronit Sharon

**Affiliations:** 1 Biochemistry and Molecular Biology, IMRIC, The Hebrew University-Hadassah Medical School, Ein Kerem, 9112001 Jerusalem, Israel; 2 Department of Neuroscience, Molecular Neurobiology, University of Oldenburg, D-26111 Oldenburg, Germany; 3 Department of Radiology, Hadassah-Hebrew University Medical Center, Ein Kerem, 9112001 Jerusalem, Israel

**Keywords:** **α-**Synuclein, Myelin, Phospholipids, Parkinson’s disease

## Abstract

α-Synuclein is a protein involved in the pathogenesis of synucleinopathies, including Parkinson’s disease (PD), dementia with Lewy bodies (DLB) and multiple system atrophy (MSA). We investigated the role of neuronal α-Syn in myelin composition and abnormalities. The phospholipid content of purified myelin was determined by ^31^P NMR in two mouse lines modeling PD, PrP-A53T α-Syn and Thy-1 wt-α-Syn. Significantly higher levels of phospholipids were detected in myelin purified from brains of these α-Syn transgenic mouse models than in control mice. Nevertheless, myelin ultrastructure appeared intact. To further investigate the effect of α-Syn on myelin abnormalities, we systematically analyzed the striatum, a brain region associated with neurodegeneration in PD. An age and disease-dependent loss of myelin basic protein (MBP) signal was detected by immunohistochemistry in striatal striosomes (patches). The age-dependent loss of MBP signal was associated with lower P25α levels in oligodendrocytes. In addition, we found that α-Syn inhibited oligodendrocyte maturation and the formation of membranous sheets in vitro. Based on these results we concluded that neuronal α-Syn is involved in the regulation and/or maintenance of myelin phospholipid. However, axonal hypomyelination in the PD models is evident only in progressive stages of the disease and associated with α-Syn toxicity.

## Introduction

The synucleinopathies are a group of neurodegenerative diseases that includes Parkinson’s disease (PD), dementia with Lewy bodies (DLB) and multiple system atrophy (MSA). These diseases share a common pathogenic insult: the accumulation of intracellular, aggregated α-synuclein (α-Syn). The occurrence of α-Syn pathology in oligodendrocytes, in the form of glial cytoplasmic inclusions (GCI) is associated with MSA, whereas accumulation of pathogenic α-Syn in neurons, in the form of Lewy bodies and Lewy neurites, is associated with PD and DLB. While α-Syn aggregation and deposition are a common denominator of these neurodegenerative diseases, the initial molecular/biochemical cause that differentiates these diseases is currently unknown.

Neurodegeneration in MSA is thought to result from loss of trophic and metabolic support provided by ensheathing oligodendrocytes [20, 67]. Myelin abnormalities and loss are a characteristic of MSA [18, 68]. Loss of structural myelin proteins, resulting in affected myelin stability, was suggested to play a causative role in the pathogenesis of MSA [61]. Similarly, lower levels of myelin lipids were shown to associate with white matter loss [15]. Importantly, myelin abnormalities, oligodendrocytic and axonal degeneration were recapitulated in mouse models for MSA. These models, overexpressing α-Syn under the CNPase promoter [71] or MBP promoter [18], demonstrated a primary oligodendroglial disease.

In contrast to MSA, PD is primarily considered a grey matter disease [57]. Importantly, neuroanatomical studies suggested that the degree of myelination, together with axonal length and axonal caliber, is a key factor determining neuronal vulnerability to Lewy pathology. Specifically, axons that develop Lewy pathology were suggested to be projections of neurons that express α-Syn and are disproportionately long, thin-caliber and sparsely or unmyelinated [5, 7–9, 50, 53].

Myelin membranes contain all major lipid groups, yet with a characteristic composition that distinguishes them from other cellular membranes [13]. The unique lipid composition of myelin is critical to its structure and function [13]. Changes in lipid composition affect lipid-protein interactions and alter membrane packing [34]. In the mouse brain, myelination of axons begins after birth and continues in adulthood, with increases in number of myelin lamellae and myelinated axons [64]. Myelin sheaths are generated throughout life by preexisting or newly formed oligodendrocytes, derived from oligodendrocyte progenitor cells (OPCs; [72]). Active myelination in the adult brain mediates a continuous myelin turnover [72]; ensures myelin remodeling that is required for learning processes [38]; and contributes to myelin repair upon demyelination under pathological conditions [26, 51]. A cross talk between oligodendrocytes and neurons determines myelin formation [4, 44, 59, 69]. However, to date, there is no known axonal signal that drives myelination of the axon that presents it (reviewed by [43]).

The striatum is a brain region associated with PD. It is responsible for the integration of motor, cognitive and emotional information into optimal behavior policy. The striatum is a complex anatomical/biochemical structure that can be differentiated into two distinct compartments: striosomes (also called patches) and matrix. Imbalances between neural activities in these two compartments are suggested to underlie the profound motor deficits observed in PD and other basal ganglia-related disorders, namely dystonia, depression and schizophrenia [14]. Importantly, striosomes and matrix differ in their input and output targets. For a long time it was accepted that striosomes preferentially project to the substantia nigra pars compacta (SNc), a brain region harboring the dopaminergic neurons that are affected in PD, whereas the matrix projects to the pars reticulata (Gerfen, 1985; Jimenez-Castellanos and Graybiel, 1989). However, a recent study has demonstrated that the predominant input to the dopamine neurons in the SNc originates outside of the striosomes and depends on the matrix, suggesting that the neurochemistry of this region is only partly understood [60].

We investigated the effect of α-Syn expression on myelin phospholipids in two mouse models for PD: PrP - A53T α-Syn [22] and Thy-1 wt α-Syn [54], in which expression of human α-Syn is driven by neuron-specific promotors. Our data provide compelling evidence that neuronal α-Syn expression increases the phospholipid content of myelin in young, non-symptomatic mice. In addition, we found evidence for myelin loss in these α-Syn tg mouse models. A systematic analysis demonstrated that myelin loss was secondary to the increases in phospholipids and was associated with α-Syn toxicity in neural and oligodendroglial cells. We conclude that the effect of neuronal α-Syn overexpression on myelin phospholipid content occurs prior to the onset of neurodegeneration.

## Methods

### Human brains

Slides with 100 μm coronal hemisphere sections from a non-PD brain (90-year-old female, NFT III/Aβ 0/PD 0) and a PD brain (68-year-old female; NFT III/Aβ 3/PD 5) stained for myelin according to a modified Pal-Weigert protocol [17] were kindly provided by the Braak laboratory (University of Ulm) for image acquisition [6, 8, 30].

### Animals

The human PrP-A53T α-Syn tg mouse line [22] was purchased from Jackson Laboratory (Bar Harbor, ME, USA) as hemizygous and cross bred with the α-Syn-/- C57BL6 mouse line (Harlan Laboratories, Jerusalem, Israel; [62]) to silence mouse α-Syn, and then bred to achieve homozygosity of the human A53T α-Syn transgene. Control mice were C57BL6 α-Syn-/- mice (Harlan Laboratories). The PrP-A53T α-Syn tg model was shown in previous studies to develop motor disabilities and to accumulate α-Syn pathology in an age-dependent manner. That is, mice appeared generally healthy and showed no evidence for α-Syn pathology up to the age of 7–8 months [22, 70]. However, at 12 months of age and older, the large majority of the mice in the colony showed pathogenic accumulations of α-Syn together with signs of motor disabilities. The number of sick mice was shown to grow with age, and the oldest mice in the colony were 16 months old. The mouse colony that we maintained fit the original description perfectly.

Thy-1 human wt α-Syn mice [54, 55] were obtained from Prof. Eliezer Masliah (UCSD, USA). Control mice were non-transgenic littermates born as a result of crossing heterozygote transgenic females with C57BL/6-DBA/2 males. The Thy-1 mouse model shows early signs of learning and motor disabilities at 2–4 months of age [21, 55] which worsen at 8–10 months of age. α-Syn pathology for the Thy-1 α-Syn mice was demonstrated at 12 months of age [55].

5XFAD mice [48] were bred and aged at Prof. Dan Frenkel’s laboratory (at Tel Aviv University). The mice carry 5 human mutations (3 in the APP and 2 in the PS1 genes) that were identified in patients affected with familial forms of Alzheimer’s disease. The mice show amyloid pathology starting at the age of 4 months and cognitive behavior impairment starting at 6 months.

Mice were housed in a 12 h dark/light cycle and were allowed free access to food and water. This study was carried out in strict accordance with the recommendations in the Guide for the Care and Use of Laboratory Animals of the National Institutes of Health. Adequate measures were taken to minimize pain and suffering. All animal welfare and experimental protocols were approved by the Committee for the Ethics of Animal Experiments of the Hebrew University of Jerusalem NIH approval # OPRR-A01-5011 (Permit number: MD-16-14826-3).

### Cultured oligodendrocytes

Oligodendrocytes were prepared as previously described [25]. Briefly, primary cultures of glial cells were prepared from the brains of newborn Wistar rats and oligodendrocytes were mechanically removed by shaking after 10–14 days in culture. Oligodendrocyte precursor cells were re-plated (1.2x10^6^ cells/6 cm dish) on poly-L-lysine (PLL)-coated culture dishes (1.2x10^6^ cells/6 cm dish) supplemented with glass cover slips (Fisher Scientific, Schwerte, Germany). Cells were grown in serum-free DMEM (Gibco/BRL, Grand Island, NY, USA), supplemented with 2 mM glutamine, 50 U/ml penicillin, 50 μg/ml streptomycin, 5 μg/ml insulin, 5 μg/ml transferrin, and 5 ng/ml sodium selenite (Roche Diagnostics, Mannheim, Germany) at 10% CO_2_. Two hours after seeding, when cells were attached to the culture dishes, medium was replaced and recombinant human α-Syn (10 μg/ml, prepared as previously described, [29]) was added. Cells were incubated for 3–6 days as indicated.

#### Western blot analysis

Cellular monolayers of control and treated cells were washed once with PBS, scraped off in sample buffer containing 1% SDS, and boiled for 10 min. Protein content in the samples was determined according to the protocol of Neuhoff, Philipp, Zimmer and Mesecke [45]. Total cellular extracts (10–30 μg protein per lane) were separated by one-dimensional sodium dodecylsulfate-polyacrylamide gel electrophoresis (SDS-PAGE) using 8.75–11.25% polyacrylamide gels and transferred to nitrocellulose membranes (Whatman, Dassel, Germany; 0.2 μm). The blots were saturated with TBS (20 mM Tris–HCl, 136.8 mM NaCl, pH 7.5) containing 5% dry milk and incubated with the indicated antibodies overnight at 4 °C. After washing with TBS-T (TBS with 0.1% v/v Tween 20), incubation with HRP-conjugated anti-mouse (1:10,000) or anti-rabbit (1:10,000) antibody was carried out for 1 h at room temperature. After washing with TBS-T, blots were visualized by the enhanced chemiluminescence (ECL) procedure as described by the manufacturer (Thermo Scientific, Rockford, IL, USA). All experiments were carried out at least 3 times with similar results. The following antibodies were used at the indicated working dilutions (in parentheses): mouse mAb anti-α-tubulin (1:1,000) and mouse mAb anti-acetylated α-tubulin (1:1,000) were from Sigma-Aldrich (Munich, Germany). Rabbit pAb anti-myelin basic protein (MBP, 1:1,000) was a generous gift from Dr. Jean-Marie Matthieu (University Lausanne, Switzerland). Mouse mAb against the chondroitin sulfate proteoglycan NG2 (Millipore, Billerica, MA, USA; 1:200). HRP-conjugated anti-mouse IgG (1:10,000) and anti-rabbit IgG (1:10,000) were from Jackson ImmunoResearch (West Grove, PA, USA).

#### Immunocytochemistry

Primary oligodendrocytes (1.2 × 10^6^ cells/6 cm dish) were cultured on PLL-coated glass coverslips in DMEM. After washing with PBS, cells were fixed and permeabilized with ice-cold methanol for 7 min. Cells were washed three times with PBS and then incubated overnight at 4 °C with the following primary antibodies: mouse mAb anti-NG2 (1:200), mouse mAb anti-α-tubulin (1:250), mouse mAb anti-acetylated α-tubulin (1:250), rabbit pAb anti-myelin basic protein (MBP; 1:200). After washing with PBS, cells were incubated for 1 h with Dylight 594-conjugated (1:500), Dylight 488-conjugated (1:500), or Dylight 350-conjugated (1:100) goat secondary antibodies (Thermo Scientific), washed with PBS, and mounted. Nuclei were stained by 4′, 6-diamidino-2-phenylindole (DAPI; 1.5 μg/ml) included in the mounting medium (Vectashield, Vector Laboratories, Burlingame, CA, USA). Fluorescent labeling was studied using a Zeiss epifluorescence microscope (Oberkochen, Germany) equipped with a digital camera using a plan-neofluar objective (x100). All experiments were carried out at least 3 times with similar results.

### Myelin purification from mouse brains and analyses

Myelin purification was done as described previously [47]. In brief, a whole mouse brain was homogenized at 1:10 w/v by dounce homogenizer in 0.32 M sucrose containing a protease inhibitor cocktail (Sigma, Rehovot, Israel). The homogenate was applied to the top of a two-step sucrose gradient (0.32 and 0.85 M sucrose). After the gradient was centrifuged at 75,000 xg for 30 min, the interface was collected, diluted 2 times with double-distilled water (DDW) and centrifuged again at 75,000 xg for 15 min. The pellet was washed twice with DDW. Myelin was weighed, resuspended in DDW and protein concentration was determined by BCA (bicinchoninic acid assay, Ornat, Rehovot, Israel).

#### Lipid extraction

Total lipids were extracted from purified myelin preparation according to the procedure of Blight and Dyer [3]. The organic phase was removed to a clean tube and dried under a stream of N_2_. The dried organic-soluble material was dissolved in 0.4 ml [^2^H]chloroform and 0.2 ml 40 mM methanolic EDTA. 0.8–4 μmol triphenylphosphate was added to each sample for quantification of the phospholipids. Methanolic EDTA consists of 200 mM EDTA in water, adjusted to pH 6.0 with CsOH and further diluted five-fold with absolute methanol [39, 66]. The solution was then transferred to an NMR 5 mm test tube and allowed to reach phase separation overnight at 4–8 °C.

#### Western blot analysis

Myelin proteins were analysed in samples of purified myelin (see above) or in whole brain extracts. Whole mouse brain was homogenized at 10% (w/v) in 10 nM Tris–HCl, pH 7.4, 0.32 M sucrose and a protease inhibitor coctail (Sigma). Protein concentration was determined by BCA protein assay kit (Ornat). Protein samples (15 μg) were loaded on either a 13.5% or a 10% SDS-PAGE, and following electrophoresis, proteins were transferred to a PVDF membrane (Biorad, Petach Tikva, Israel). The membrane was blocked with 5% non-fat dry milk in Tris-buffered saline pH 8.0 (10 mM Tris–HCl, 150 mM NaCl, pH 8.0) containing 0.1% Tween-20 (TBST) for 1 h. The membrane was then incubated at 4 ^0^C for 16–18 h in 1% non-fat dry milk in TBST and a specific antibody. The following antibodies were used at the specified concentrations: anti-rat MBP mAb (1:1500, Serotec, Hercules, CA, USA); anti-rabbit MAG mAb (1:1000, Cell Signaling Technology, Danvers, MA, USA); anti-mouse CNPase mAb (1:500, Sigma); anti-mouse MOG mAb (1:5000, Merck-Millipore, Darmstadt, Germany); anti-rabbit PLP/DM20 pAb (1:1000, Novus Biologicals, Littleton, CO, USA); anti-rat tubulin mAb (1:10,000, AbDSerotec); and anti-P25α pAb (1:500, a gift from Paul Henning Jensen, Aarhus University, Denmark). Immunoreactive bands were detected with HRP-conjugated donkey anti-mouse (1:10,000), goat anti-rat (1:10,000), or goat anti-rabbit (1:10,000) secondary antibody. The signal was visualized with EZ-ECL (Biological Industries, Beit Haemek, Israel), scanned with a Umax Magic Scan (Eastman Kodak, Rochester, NY, USA) and analyzed for density of each signal using UN-SCAN-IT GEL 3.1 software (Silk Scientific, Orem, UT, USA). The signal obtained for each protein in a specific sample of myelin was normalized to the total amount of tubulin protein in the same sample.

#### Flotation assays

Flotation of detergent-insoluble complexes was performed as described by [42]. In brief, whole mouse brains were homogenized 1:10 w/v in an ice-cold buffer containing sodium chloride 150 mM, Tris–HCl pH 7.5 25 mM, EDTA 5 mM and 1% Triton X-100. Insoluble particles were spun down and the homogenate was loaded (60 μl/samples) into the bottom of ultracentrifuge tubes (TLS-55; Beckman Instruments, Inc, Fullerton, CA). An equal volume of ice-cold Nycodenz (Biological Industries, Beit Haemek, Israel), 70% in TNE (Tris–HCl pH 7.5 25 Mm, sodium chloride 150 mM, and EDTA 5 mM) was added and mixed with the lysate. An 8 to 25% Nycodenz linear step gradient in TNE was then overlaid above the lysate (200 μL each of 25%, 22.5%, 20%, 18%, 15%, 12%, and 8% Nycodenz). The tubes were spun at 55,000 rpm for 4 h at 4 °C in a TLS-55 rotor (200,000 xg). Fractions were collected from top to bottom of the tube. Each fraction was applied to a 13.5% sodium dodecyl sulfate-polyacrylamide gel electrophoresis (SDS-PAGE) and immunoblotted with the specified antibodies.

**NMR spectra** of the processed lipid extracts were recorded on a 500 MHz NMR spectrometer (Bruker, Germany) with a 5 mm broadband probe. The ^31^P chemical shifts were referenced to phosphatidylcholine at 0 ppm. Spectral processing was carried out using MNova (Mestrelab Research, Santiago de Compostela, Spain).

### Transmission electron microscopy

Mouse brains were removed and thick coronal sections (100 μm) were obtained using a vibrotome (Leica Biosystems, IL, USA). Brain sections were fixed in a solution containing 2% paraformaldehyde, 2.5% glutaraldehyde (EM grade) in 0.1 M sodium cacodylate buffer (pH 7.3) for 2 h at room temperature and then transferred to 4 °C for an additional 24 h. Brain sections were washed 4 times with sodium cacodylate and incubated for 1 h in 1% osmium tetroxide, 1.5% potassium ferricyanide in sodium cacodylate. Sections were then washed 4 times in the same buffer; dehydrated with graded series of ethanol solutions (30, 50, 70, 80, 90, 95%) for 10 min each; then in 100% ethanol 3 times for 20 min each; followed by 2 changes of propylene oxide. Brain sections were infiltrated with series of epoxy resin, (25, 50, 75, 100%) for 24 h each and polymerized in the oven at 60 °C for 48 h. The blocks were sectioned by an ultramicrotome (Ultracut E, Riechert-Jung, Ontario, Canada) and sections of 80 nm were stained with uranyl acetate and lead citrate. Sections were observed using a Jeol JEM 1400 Plus Transmission Electron Microscope and pictures were taken using a Gatan Orius CCD camera.

### Immunohistochemistry

Paraffin sections (6 μm) were processed for immuno-staining as previously described [36] with some modifications. For detection and quantitation of MBP, or double staining of MBP/α-Syn, slides were treated in 80% formic acid for 10 min, followed by washes and then blocked in CAS-BLOCK (Invitrogen, Carlsbad, CA, USA) for 30 min at room temperature. Slides were then incubated with anti-MBP antibody (1:200) in 1.5% BSA in 0.1 M Tris–HCl pH 7.6 containing 0.3% Triton-X100, followed by Alexa Fluor 647-conjugated donkey anti-rat IgG (1:100, Jackson Laboratories). α-Syn detection was performed using anti-mouse α-Syn, Syn303 (1:3000, from Prof. Virginia M.-Y. Lee, Philadelphia, PA, USA) and Alexa Fluor 488-conjugated goat anti-mouse IgG (1:200, Jackson Laboratories). For detection of TH antigen retrieval was done in 10 mM citrate buffer, pH 6.0 at 110 ^0^C (Decloaking Chamber, DC2012, Biocare Medical, Concord, CA, USA) for 15 min. Slides were blocked in CAS-BLOCK for 10 min at RT, reacted with anti-mouse TH (1:3000, Sigma) antibody and Alexa Fluor 488-conjugated goat anti-mouse IgG (1:200, Jackson Laboratories). P25α staining was done following pre-treament of slides in 10 mM citrate buffer, pH 6.0 at 95 ^0^C for 15 min. Blocking in 5% normal goat serum (Jackson Laboratories) in 0.1 M Tris–HCl pH 7.6 containing 0.3% Triton-100, for 2 h at RT. Immunoreaction with anti-rabbit P25α antibody (1:50, a gift from Paul Henning Jensen, Aarhus University, Denmark) and Cy2-conjugated goat anti-rabbit (1:100, Jackson Laboratories). Images were acquired using a Zeiss LSM 710 Axio Observer confocal Z1 laser scanning microscope, equipped with an argon laser 488, Diode 405-30 laser and HeNe 633 laser. The fluorescence was collected by employing a Plan-apochromat 25x/0.8 W oil/glicerin DIC (Zeiss). Per each experiment, exciting laser, intensity, background levels, photo multiplier tube (PMT) gain, contrast and electronic zoom were maintained at the same level. For each antibody, the background was subtracted (determined by a negative control consisting of the secondary antibody alone). The zoom of each picture was obtained by choosing the plane with greatest fluorescent signal.

### α-Syn pathology

Histochemical analysis was performed as described previously [70]. Briefly, sections of 6 μm were deparaffinized in xylene followed by graded alcohol in descending ethanol concentrations. Endogenous peroxidase activity was inhibited by incubation in methanol/H_2_O_2_ (150 ml methanol and 30 ml of 30% H_2_O_2_). Antigen retrieval was performed by incubating the slides in 100% formic acid for 5 min followed by extensive washes. The sections were blocked in 2% fetal bovine serum in 0.1 M Tris–HCl, pH 7.6. Sections were then immunostained using anti-α-Syn antibody Syn-303 (1:3000; a gift from Prof. Virginia M.-Y. Lee). Secondary antibody was biotinylated donkey anti-mouse (1:200; Enco Petach Tikvah, Israel), followed by ExtrAvidin (Sigma; 1:100 in blocking solution). Immunoreactivity was visualized diaminobenzidine as chromogen (Zymed Laboratories Inc.).

### Luxol Fast Blue staining

Sections of mouse brains (6 μm) were deparaffinized in xylene and hydrated in descending ethanol concentrations. Sections were then incubated in 0.1% Luxol Fast Blue Solution (Sigma) at 56 ^0^C for 16–18 h, allowed to cool at RT, rinsed for 5 min in 95% ethanol in distilled water to remove excess blue stain. Sections were subsequently placed in 0.1% lithium carbonate aqueous solution for 2 min, dipped several times in 70% ethanol in distilled water in order to stop the reaction. Sections were then transferred to a chamber containing 0.8% periodic acid solution (Sigma) for 10 min and finally stained in Schiff’s reagent for 20 min, followed by 3 washes in a buffer containing 0.5% HCL (vol/vol) and 0.2% potassium disulfite (w/vol) for 2 min each. Sections were mounted with coverslips and then observed using light microscopy.

### Quantification

Quantifications were performed blindly to treatments. To reduce experimental error, quantifications were performed in sets of four slides, representing each of the groups and comparisons were made only between slides of comparable brain regions (according to H&E staining) that were stained and handled in parallel. Image series were analyzed with Image Pro Plus 6.3 (Media Cybernetics, Bethesda, MD, USA). An average value was calculated for each animal, followed by calculation of the group average values (± SD). To sum up the different comparisons and compare between the different staining events, we present the data as a percent of the control mice, which were set at 100% for each staining event.

### Statistical analysis

Multiple comparisons between groups were done using one-way ANOVA.

## Results

### Higher levels of phospholipids in purified myelin from α-Syn tg mouse brains

Myelin phospholipid composition was analyzed in brains of A53T α-Syn tg mice using ^31^P NMR spectroscopy. In this method, the phospholipids are detected and quantified in a lipid extract with no need for multiple purification steps. Mice were analyzed at 4–6 months of age when they are fully myelinated [64]; show no evidence for behavioral or motor abnormalities [22]; and no evidence for α-Syn pathology [22, 70]. Myelin was purified from whole mouse brains and total lipids were extracted from the purified myelin by chloroform/methanol (see methods). The phospholipid resonances were obtained within a chemical shift range of about 1.2 ppm. Assignment of the resonances in the NMR profile was done based on standard phospholipids spiked into the sample and according to previous assignments performed under similar solvent conditions [16, 32]. The phosphorous signals for standard phosphatidic acid (PA) and phosphatidyl serine (PS), spiked into a chloroform/methanol extract of a myelin sample, were identified at 1.10–1.15 and 0.72–0.78 ppm, respectively.

**Fig. 1:**
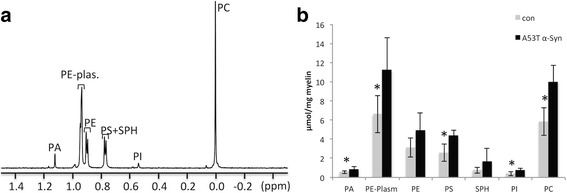
Higher levels of phospholipids in purified myelin from neuronal α-Syn-expressing mouse brains. **a** ^31^P NMR spectra of a sample consisting of a chloroform/methanol extract of purified myelin obtained from a Thy-1 human wt α-Syn tg mouse brain. The ^31^P NMR spectra were obtained with a 500 MHz NMR spectrometer (Bruker, Germany) with a 5 mm broadband probe. The ^31^P chemical shifts were assigned according to standard phosphatidic acid (PA) and phosphatidylserine (PS), spiked into the sample and referenced to phosphatidylcholine (PC) pick at 0 ppm. **b** The area under the curves was calculated for samples of A53T α-Syn and control mouse brains and the relative amount of specific phospholipids was calculated using the MNova data processing program (Mestrelab Research); mean ± SD of *n* = 5 mouse brains; *, < 0.05, one-way ANOVA. phosphatidylethanolamine plasmalogen (PE-plasm); phosphatidylethanolamine (PE); sphingomyelin (SPH); phosphatidylinositol (PI); phosphatidylcholin (PC)

The mean total phosphorous signal detected in samples of A53T α-Syn was found to be 36.4 ± 7.5 μmole per mg myelin and was significantly higher than the signal determined in control brains (19.9 ± 5.8 μmole/mg myelin, *n* = 5 brains, *p* < 0.05, one-way ANOVA). Using a data processing program (MNova) the area under the curves was determined and the relative amount of specific phospholipids was calculated. Significantly higher levels of PA, PC, PI, PS and PE-plasmalogen were calculated for A53T α-Syn than for control mouse brains. In contrast, the increases in levels of PE and SPH in the A53T α-Syn brains were not significant (Table 1 and Fig. 1a,b). Importantly, total protein levels in purified myelin preparations were closely similar between control mouse brains (0.814 ± 183 mg protein) and A53T α-Syn tg mouse brains (0.97 ± 225 mg protein).

**Table 1:** Levels of phospholipids detected by ^31^P NMR in myelin purified from whole A53T α-Syn and control mouse brains

	Control	A53T α-Syn	*P*
PA	0.56 ± 0.16	0.90 ± 0.08	0.02*
PC	5.8 ± 1.4	10.5 ± 1.6	0.02*
PE	3.1 ± 1.0	4.9 ± 1.8	0.06
PI	0.4 ± 0.2	0.8 ± 0.2	0.02*
PS	2.6 ± 0.9	4.6 ± 0.5	0.05*
PE-plasm	6.6 ± 1.9	11.5 ± 3.3	0.03*
SPH	0.8 ± 0.3	1.7 ± 1.1	0.12

Table 1 Calculated phosphorus signals for assigned phospholipids detected in lipid extracts of purified myelin from whole A53T α-Syn and control mice (in μmol per mg purified myelin, *n* = 5 mice). *PA* phosphatidic acid, *PC* phosphatidylcholine, *PE* phosphatidylethanolamine, *PI* phosphatidylinositol, *PS* phosphatidylserine, *SPH* sphingomyelin; and PE-plasm., phosphatidylethanolamine-plasmalogen; *P < 0.05, one-way ANOVA

In order to confirm the effect of α-Syn overexpression on the phospholipid content of myelin, we employed a second mouse model, Thy-1 human wt α-Syn transgenic mice. Mice were analyzed at 4–5 months of age (*n* = 4). The analysis indicated highly similar results. That is, a significant ~17% higher phosphorous signal was detected in lipid extracts of myelin purified from Thy-1 tg than control mouse brains. Importantly, the levels of PC, PE-plasmalogen and PI were 10–22% higher in the Thy-1 human wt α-Syn brains. Together, the ^31^P NMR analyses indicated significantly higher contents of phospholipids in samples of purified myelin obtained from α-Syn overexpressing mouse brains than control mouse brains.

### Most of the tested myelin proteins showed unaffected levels

**Fig. 2:**
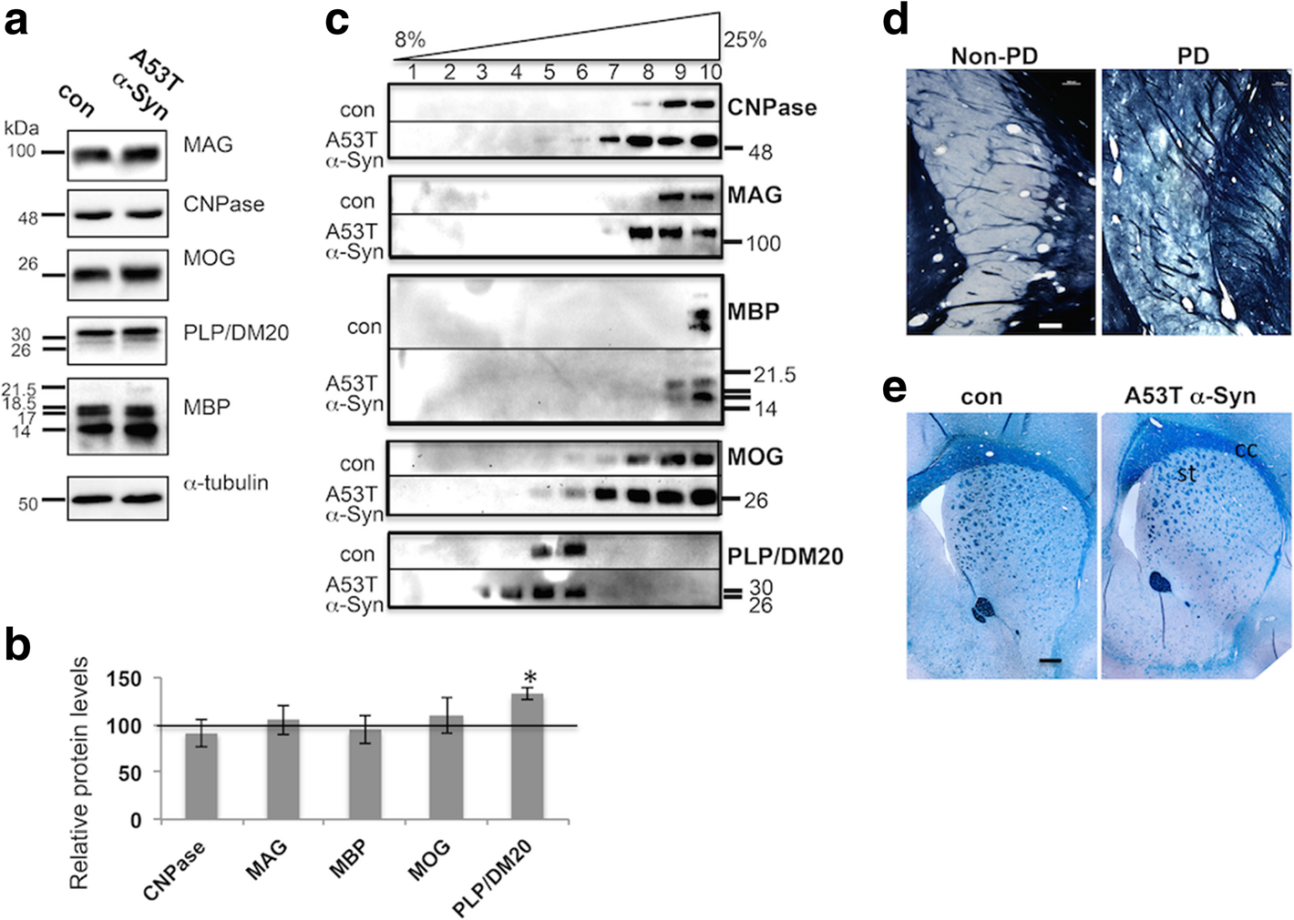
Myelin proteins, membrane flotation and histology. **a** Western blot showing myelin proteins in preparations of purified myelin from A53T α-Syn and control mouse brains at 4–6 months of age (*n* = 5–6 mice). **b** Bars showing mean ± SD of the indicated myelin protein levels, in whole brain protein extracts, of A53T α-Syn at 12–14 months (*n* = 8–9 mice). Presented as a percent of control age-matched mouse brain, set at 100% (vertical line) *, < 0.05, one-way ANOVA. **c** Flotation assay showing the distribution of detergent-soluble myelin membrane particles into a 8–25% nycodenz gradient. Purified myelin preparations of A53T α-Syn and control mouse brains at 12 months of age, analyzed in parallel. Aliquots of gradient fractions analyzed by Western blotting using the specified antibodies. Representative blot of *n* = 3. 2', 3'-cyclic nucleotide 3'-phosphodiesterase (CNPase); myelin associated glycoprotein (MAG); myelin basic protein (MBP); myelin oligodendrocyte glycoprotein (MOG); proteolipid protein (PLP). **d** The caudate nucleus in coronally sectioned human brain hemisphere (100 μm), including striosomes (dark strips) and matrix (light staining) of a 90-year old female without PD (non-PD) and a 68-year-old female PD patient with advanced disease (neuropathological stage 5). Sections were stained for myelin using a modified Pal-Weigert method. Scale bar, 500 μm. Stained brain sections were provided by the Braak laboratory (University of Ulm, Ulm, Germany). **e** Coronal brain sections (6 μm, paraffin embedded) of A53T α-Syn and control mice at 12 months of age stained with Luxol Fast Blue/Periodic Acid Schiff, showing the corpus callosum (cc) and striatum (st). Scale bar, 500 μm

Myelin membranes consist of myelin-specific proteins, which are critical for myelin structure, assembly and integrity, because they regulate the molecular organization within the myelin sheath [40]. The levels of specific myelin and oligodendroglial proteins were determined in preparations of purified myelin by quantitative Western blotting, using specific antibodies. Myelin was purified from whole brains of A53T α-Syn or control mice at 4–6 months of age. The levels of myelin basic protein (MBP) and proteolipid protein (PLP), which together constitute about 70% of myelin protein, were determined, as were those of 2′, 3′-cyclic nucleotide 3′-phosphodiesterase (CNPase), myelin oligodendrocyte glycoprotein (MOG), and myelin-associated glycoprotein (MAG). This protein analysis indicated no significant differences in levels of CNPase, MAG, MBP, MOG or PLP proteins between the indicated mouse lines at the age of 4–6 months (Fig. 2a, *n* = 5–6 mouse brains). Similar results, indicating no significant differences in levels of the specified myelin proteins, were detected by Western blotting of whole mouse brain extracts of 4–6 month-old A53T α-Syn and control mouse brains, suggesting that the increases in levels of phospholipid (above) are not associated with corresponding changes in levels of myelin protein in young and healthy mice.

We next determined the levels of myelin proteins in preperations of purified myelin from whole brains of symptomatic 12–14 month-old mice (*n* = 8–9 mice). Similar to the 4–6 month-old mice, the analysis of the symptomatic 12–14 month old mice showed closely similar levels of CNPase, MAG, MOG and MBP to the levels detected in control mouse brains. However, a significantly higher PLP level was detected in the A53T myelin preparations. That is, when the PLP signal obtained in control mice was set to 100%, the PLP signal in old A53T α-Syn mice was 132 ± 12% (Fig. 2b).

Together, the quantitative analysis of myelin proteins indicated that myelin protein levels were basically unaffected, with the exception of PLP protein levels, which were higher in old, symptomatic mouse brains.

### Flotation assays suggest altered protein/lipid ratio in myelin membranes

Membrane flotation on a nycodenz gradient is determined by the membrane’s lipid and protein content. In general, membranes associated with lighter gradient fractions have a higher ratio of lipids to proteins than those associated with heavier gradient fractions. We examined whether the increases in myelin phospholipids affect myelin flotation on a nycodenz gradient. We purified myelin from 12 month-old A53T α-Syn and age-matched control mouse brains; solubilized the membranes with Triton X-100; and loaded into the bottom of a linear gradient (8 to 25% nycodenz). Following centrifugation, gradient fractions were collected from top to bottom (fractions 1–10, respectively); flotation was analyzed by Western blot using specific antibodies against CNPase, MAG, MBP, MOG and PLP. We found a mild yet consistent shift of myelin proteins toward lighter fractions on the A53T α-Syn gradient compared to control mouse brains (Fig. 2c). A shift of 1–2 fractions was observed for CNPase, MAG, MBP, MOG and PLP proteins. This result may support a higher content of lipids in these membranes.

### No obvious indications for hypomyelination in human brains with PD or neuronal-derived α-Syn-expressing mouse models

The architecture of the striatum differs between human and mouse brains [73]. Whereas the human brain striatum is separated into caudate and putamen by the internal capsule, the mouse striatum lacks this architectural division. Nevertheless, the organization of the striatum into sub-compartments, including the matrix, which is sparsely myelinated, and striosomes, which are highly myelinated, is preserved in the human and mouse striatum. Importantly, no obvious differences were evident between the myelin content in the striatum of a PD case (stage 5) and a control case, negative for α-Syn pathology (Fig. 2d).

To visualize myelin in mouse brains, we stained paraffin-embedded brain sections with Luxol fast blue. A similar staining pattern was detected for 12–14 month-old A53T α-Syn and age-matched control brain sections, with no evidence for obvious loss of myelin (Fig. 2e). A similar result was also obtained for 9–10 month-old Thy-1 α-Syn tg brains (not shown). The corpus callosum, which represents a brain region enriched with white matter, appeared intact with no signs of hypomyelination (Fig. 2e). We concluded that, similar to the situation in human brains with PD, there is no gross or obvious myelin loss in the mouse models for PD.

### Myelin ultrastructure

**Fig. 3:**
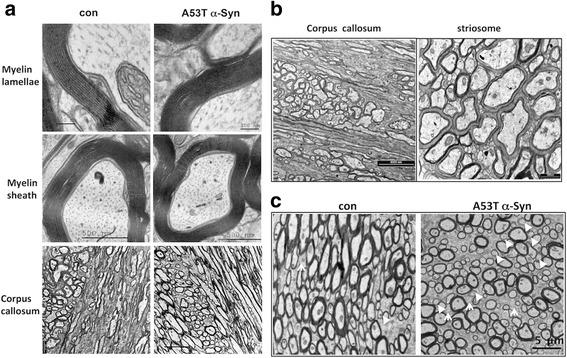
Myelin ultrastructure. **a** Representative electron micrographs of myelin ultrastructure in 12 month-old A53T α-Syn and age-matched control mouse brains. Coronal sections were prepared from a brain region containing the corpus callosum and rostro-dorsal striatum (with the size of the lateral ventricle as a reference for section position). **b** Electron micrographs as in (**a**) showing corpus callosum and striosome of a 5 month-old A53T α-Syn tg mouse brain. **c** Electron micrographs of striosomes in 12 month-old A53T and age-matched control brains. Arrow, point at sparsely myelinated axons

To find out whether the higher phospholipid levels measured in myelin may affect myelin ultrastructure, we analyzed A53T α-Syn and control mouse brains at 5 and 12 months of age by transmission electron microscopy (TEM). Cross sections containing the corpus callosum and striosomes revealed intact myelin with no ultrastructural abnormalities. At both ages tested, the majority of axons in the striosomes of A53T α-Syn (Fig. 3a, b) were surrounded by myelin sheath, with a standard number of lamella and basically unaffected myelin thickness. In addition, the ultrastructure analysis indicated intact myelin in the corpus callosum of 5 month-old (Fig. 3b) and 12 month-old (Fig. 3a) A53T α-Syn tg mouse brains. A few unmyelinated axons were detected in striosomes of A53T α-Syn at 5 months of age (Fig. 3b). However, a higher number of un-myelinated and sparsely myelinated axons was detected in the striosomes of 12 month-old A53T α-Syn than in control mouse brains (Fig. 3c, white arrows). These axons appeared to have a periaxonal cytoplasmic collar with a poor myelin sheath. In addition, many unmyelinated small-sized axons were in direct contact with each other, without interdigitating glial processes (Fig. 3c).

### Localized MBP loss occurred in an age- and disease-dependent manner

**Fig. 4:**
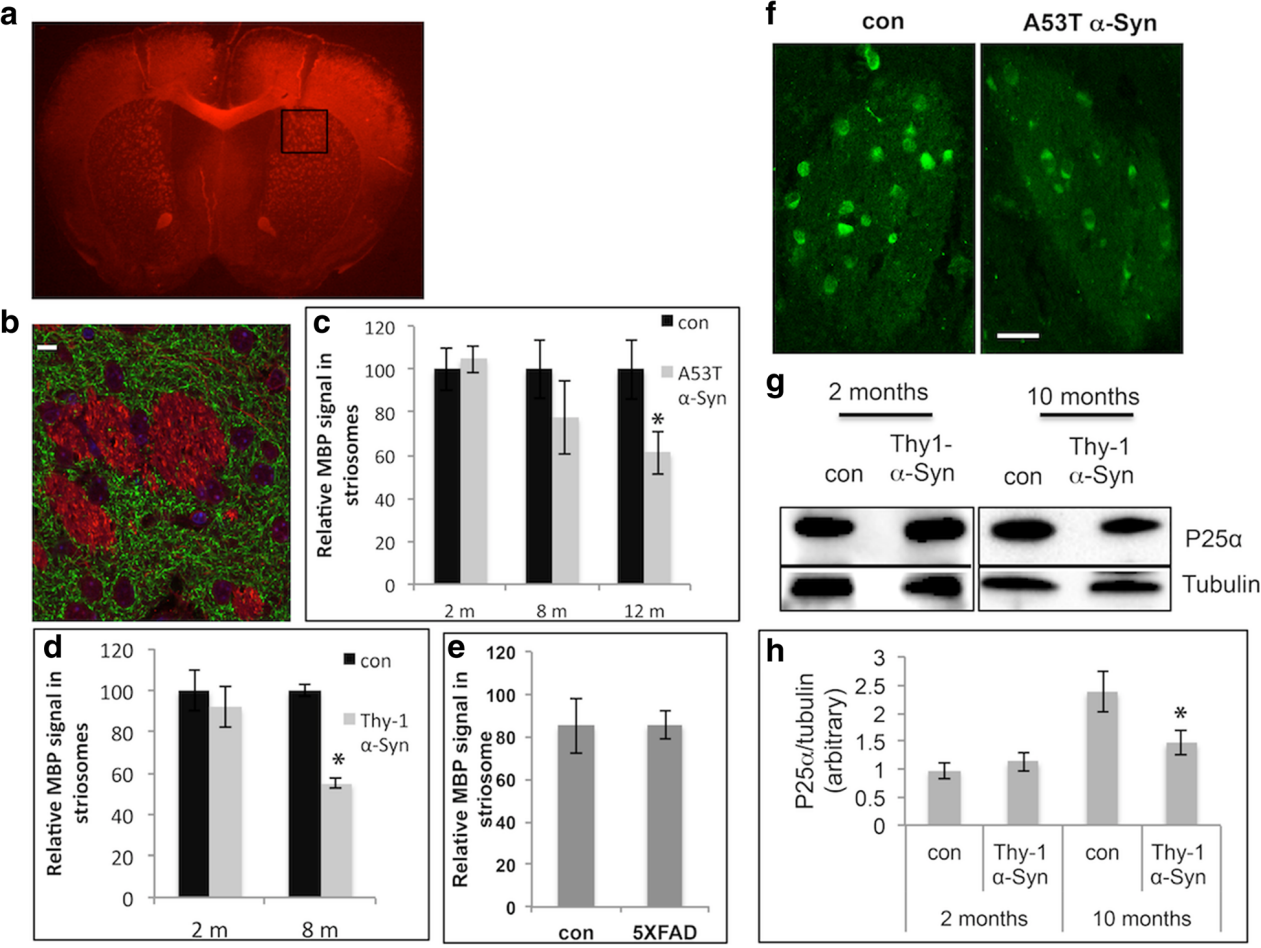
Age-dependent loss of MBP and P25α signals in striosomes (patches). **a** A coronal brain section (6 μm, paraffin embedded) from a 12 month-old A53T α-Syn mouse, stained with anti-MBP antibody. Black square shows area of interest. **b** Higher magnification of the area of interest from (**a**) in a consecutive section, double stained with anti-MBP (*red*) and anti-tyrosine hydroxylase (TH, *green*) antibodies. Scale bar, 100 μm. **c** Quantification of MBP signal inside striosomes. Bars represent mean ± SD of *n* = 4 A53T and control mice at 2, 8 and 12 months of age. *, < 0.05, one-way ANOVA. **d** Quantification of MBP signal in striosomes of Thy-1 α-Syn tg and age-matched control mouse brains determined at 2 and 8 months of age. Bars represent mean ± SD of *n* = 4 mice. *, < 0.05, one-way ANOVA. **e** Quantification of MBP signal in striosomes of 12 month-old 5XFAD and control mice. **f** Coronal brain sections containing rostral-dorsal striatum (6 μm, paraffin embedded) of 12 month-old A53T α-Syn mouse and age-matched control immunoreacted with anti-P25α antibody and showing a striosome. Scale bar, 10 μm. **g** Samples of high-speed supernatant (50 μg protein) obtained from whole Thy-1 α-Syn and control mouse brains at 2 and 10 months of age analyzed by Western blotting and immunoreacted with anti-P25α antibody. **h** Graph showing quantitation of blots obtained in (G) mean ± SD of *n* = 4 mice. *, < 0.05, one-way ANOVA

To quantify myelination in striosomal axons, we systematically stained brain sections from A53T α-Syn and control mice for MBP using inmunohistochemistry, and focused on the striatum, including matrix and striosomes. A faint MBP signal was observed in the striatal matrix, whereas a strong MBP signal was observed in the striosomes. We specifically focused on the dorsal/caudal striatum, immediately underneath the corpus callosum, using the size of the lateral ventricle as a reference for the location of the brain slice (Fig. 4a). MBP signal in axons of striosomes is easily distinguished from axons in matrix, which are immunoreactive to thyrosine hydroxylase (TH; Fig. 4b). The striosomal MBP signal was systematically quantified in brains of A53T α-Syn tg mice at 2, 8 and 12–14 months of age (*n* = 4–5 brains for each genotype and each age group; Fig. 4c).

There were no significant differences in MBP levels in the striosomes of A53T α-Syn tg and control mice at the young age of 2 months. At 8 months, we detected a tendency towards a lower MBP signal in the A53T α-Syn brains; however, heterogeneity in the measured values denied significancy. At 12–14 months of age, the MBP signal was significantly lower in striosomes of A53T α-Syn tg mice than in control mice. Setting the MBP signal detected in striosomes of control mice to 100%, A53T α-Syn brains had a relative MBP signal of 61.26 ± 15.5% (*p* < 0.01, one-way ANOVA). A similar systematic analysis of MBP signal was performed for Thy-1 α-Syn tg mice, with highly similar results (Fig. 4d). Specifically, no significant differences were detected at 2 months of age. However, a significantly lower MBP signal was detected in striosomes of Thy-1 α-Syn tg mice at 8–9 months of age. Relative to the control mice, Thy-1 α-Syn mice had an MBP signal of 55.1 ± 2.7% (mean ± SD, *n* = 4 mice in each genotype, *p* < 0.02, one-way ANOVA). Since lower MBP levels were detected at 8–9 months, we did not continue the analysis at 12–14 month for Thy-1 α-Syn mice.

MBP signal was quantified similarly in striosomes of a mouse model for Alzheimer’s disease, 5X FAD (Fig. 4e). Importantly, no differences in MBP signal in striosomes were found between the 5X FAD and control mice at 12 months of age. Together, the results show an α-Syn-specific and age-dependent effect on myelin signal in striosomes.

### Lower P25α levels in oligodendrocytes of A53T α-Syn tg mouse brains

To find out whether oligodendrocytes are affected in A53T α-Syn tg mice, we stained mouse brain sections for P25α, a marker for myelinating oligodendrocytes. The number of oligodendrocytes positively stained with P25α was similar in striosomes of A53T α-Syn and control mouse brains at 2 months (not shown) and 12 months of age (*n* = 4; Fig. 4f). In addition, similar numbers of oligodendrocytes were detected in the corpus callosum of A53T α-Syn and control mice (not shown). No evidence for accumulation of P25α in cytoplasmic inclusions was detected in brain sections of symptomatic 12–14 month-old A53T α-Syn mice. The occurrence of P25α in oligodendrocytes appeared throughout the cytoplasm and also in the nucleus (Fig. 4f). Importantly, a lower P25α signal was detected in the 12 month-old A53T α-Syn oligodendrocytes. That is, setting the signal obtained for P25α in striosomes of control brains to 100%, we detected a relative signal of 72.4 ± 12.2% in corresponding brain sections from A53T α-Syn tg mice (*n* = 4 brains; 12–14 striosomes in each genotype; *P* < 0.05, one-way ANOVA). A similar lower P25α signal was detected in the corpus callosum of 12 month-old A53T α-Syn mouse brains (not shown).

Next, we determined P25α levels by Western blotting. The striatum was removed from Thy-1 α-Syn and control mouse brains at 2 months or 10 months of age. The soluble fraction was analyzed using an anti-P25α antibody. The results showed an age-dependent effect on P25α levels. That is, P25α levels were highly similar in Thy-1 α-Syn and control mice at 2 months of age, but P25α levels were ~39% lower in the 10 month-old Thy-1 α-Syn than control mouse brains (*n* = 4, Fig. 4 g,h). Together, the results obtained with P25α staining showed no evidence for oligodendrocytic pathology in the form of inclusion formation or cell loss; however, the lower P25α signal detected in old α-Syn tg mouse brains suggested an age- and disease-dependent effect on oligodendrocyte activity.

### α-Syn uptake delayed maturation and myelin-like membrane formation in mature primary oligodendrocytes

**Fig. 5:**
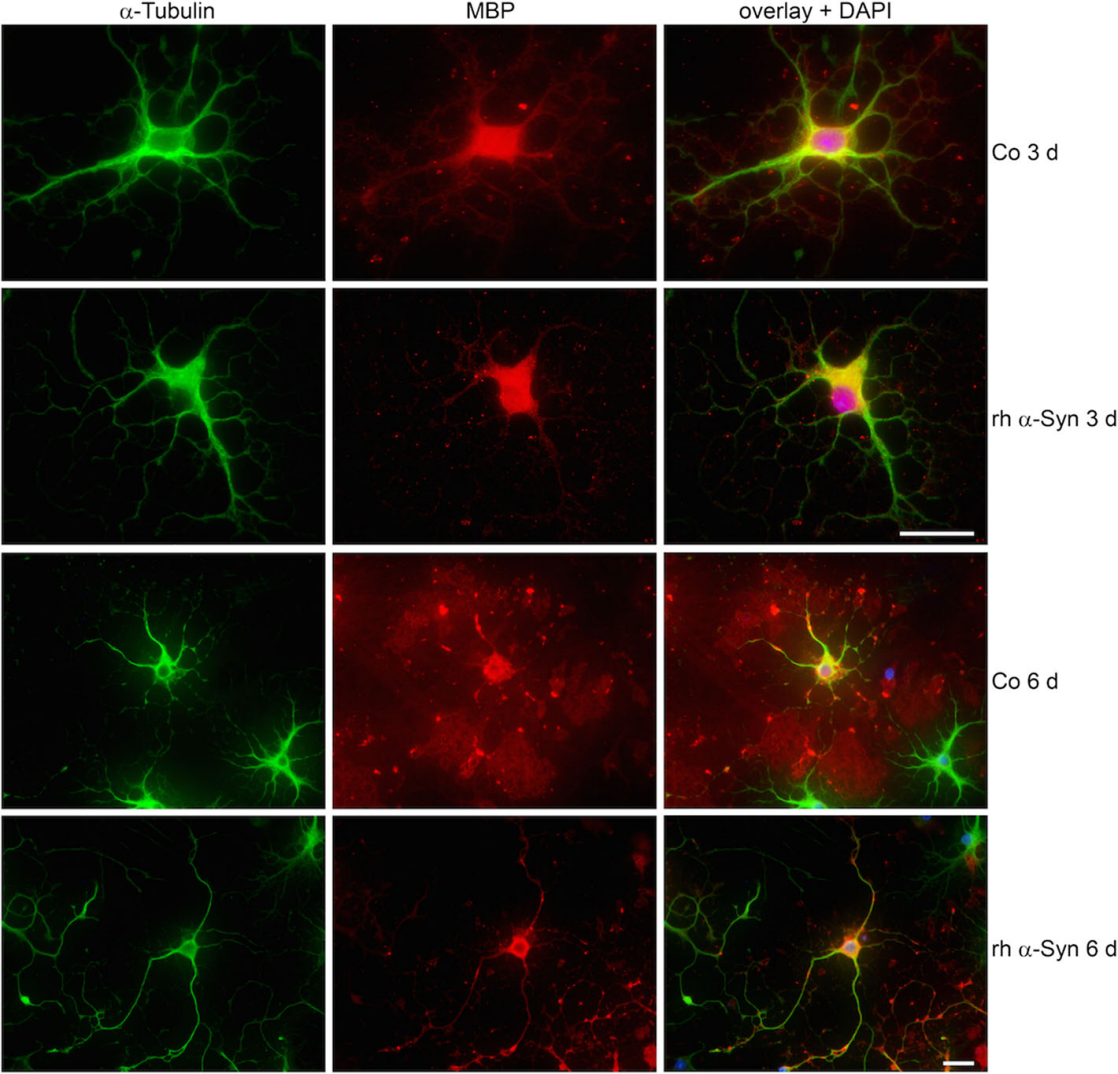
Effects of α-Syn on oligodendrocyte differentiation. Oligodendrocyte progenitor cells were either untreated (Co) or incubated with recombinant human (rh)α-Syn (10 μg/ml) 2 h after plating for the indicated time. Cells were subjected to indirect immunofluorescence staining using antibodies against α-tubulin (*green*) and MBP (*red*). Nuclei were stained with DAPI (*blue*). Scale bar: 20 μm

**Fig. 6:**
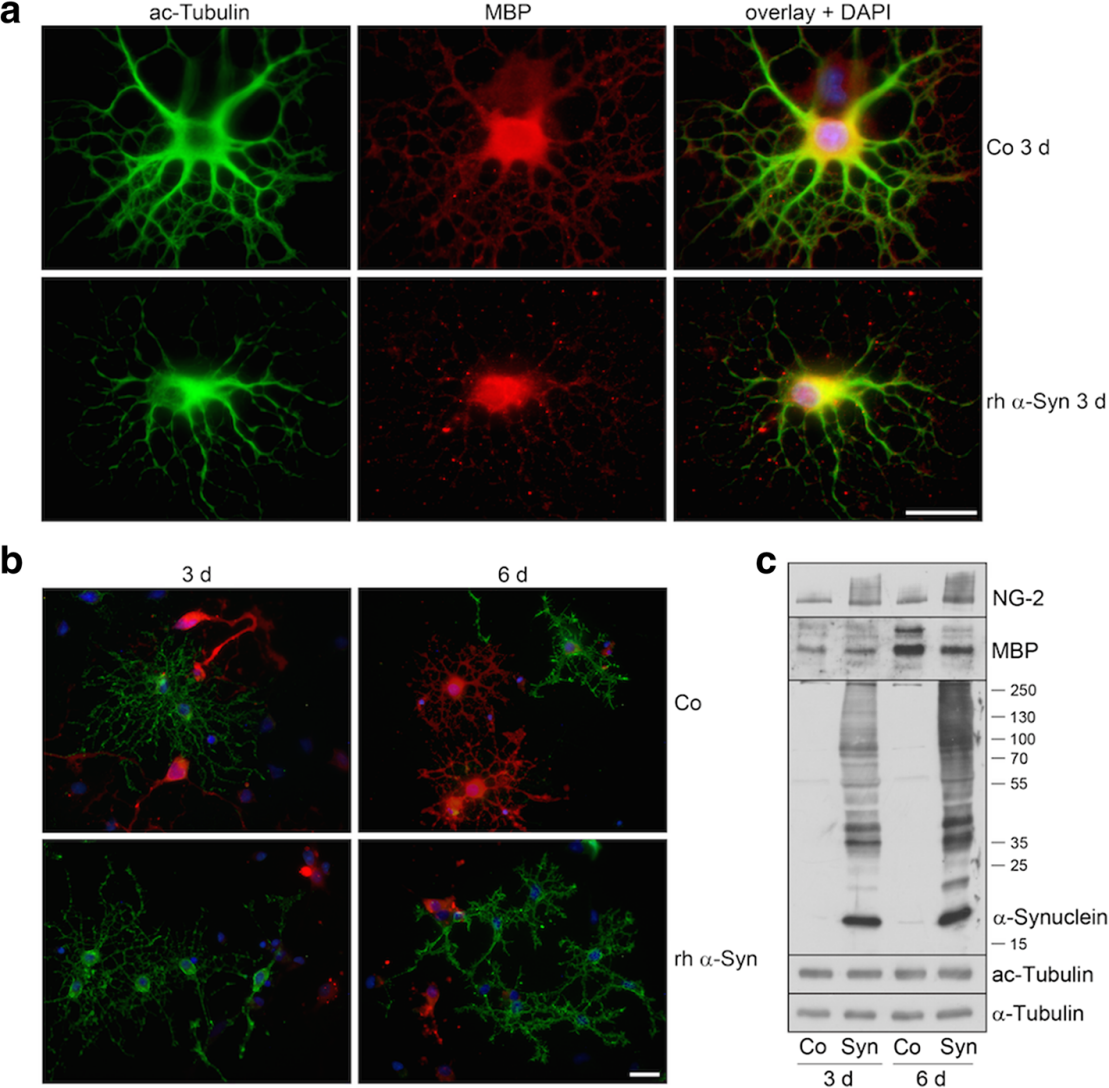
α-Syn impairs oligodendrocyte maturation. Oligodendrocyte progenitor cells were either untreated (Co) or incubated with rh α-Syn (10 μg/ml) 2 h after plating for 3 or 6 days. Cells were subjected to immunocytochemistry using antibodies: **a** anti-acetylated α-tubulin (*green*) and anti-MBP (*red*); **b** anti-proteoglycan NG-2 (*green*) and anti-MBP (*red*). Nuclei were stained with DAPI (*blue*). Scale bar: 20 μm. **c** Exogenously applied α-Syn led to an increase in NG-2 and a decrease in MBP levels. Western blot analysis of cell extracts was carried out with antibodies indicated on the right. Numbers on the right indicate molecular weights in kDa

In previous studies, we have shown that primary mature oligodendrocytes are capable of taking up α-Syn from their environment. Moreover, α-Syn uptake does not exert cytotoxic effects *per se*, yet, under stress conditions, for example, oxidative stress, larger α-Syn aggregates are formed which may eventually contribute to cytotoxicity and cell death [33, 52]. Here we tested whether α-Syn uptake affects oligodendrocyte maturation and myelin-like membrane formation *in vitro*. To this end, oligodendrocyte precursor cells were prepared and two hours after seeding, when they had attached to the cell culture surface, were subjected to recombinant human α-Syn (10 μg rh-α-Syn/ml medium). Immunocytochemistry (ICC), using antibodies against MBP and α-tubulin, revealed that cell differentiation was impaired (Fig. 5). After three days in culture with rh-α-Syn, MBP expression was rather low and cellular processes were not as elaborate as in control cells, conditioned in parallel, but without rh-α-Syn. This effect was even more pronounced after 6 days of treatment: cells did not extend flat membranous MBP-positive sheets, and the microtubule-containing arborizations appeared less bundled and rather thin (Fig. 5). However, microtubules were positively stained with antibodies against acetylated α-tubulin (ac-tubulin) throughout the cell body and the processes (Fig. 6a). Hence cells appeared to be impaired in their development but with no signs of degeneration. The effect of α-Syn uptake to slow down the development of cultured oligodendrocytes was further corroborated by ICC staining with antibodies against NG-2, a chondroitin sulfate proteoglycan, which represents a marker for oligodendrocyte precursor cells [46, 65]. Figure 6b shows that in the presence of rhα-Syn, NG-2 positive cells were prominent in the cultures during *in vitro* differentiation, while MBP-positive cells were scarcely present and morphologically not as arborized as in the control cultures. A Western blot analysis of samples of oligodendrocyte extracts supported this notion. As demonstrated in Fig. 6c, after treatment with rh-α-Syn for 3 and 6 days, lower MBP levels were detected and the levels NG-2 were enhanced, while no change in the total amount of α-tubulin or ac-tubulin was observed. Thus, oligodendrocyte precursor cells react to the uptake of α-Syn and their cellular differentiation is impaired.

### Age-dependent accumulation of α-Syn pathology in the striatum

**Fig. 7:**
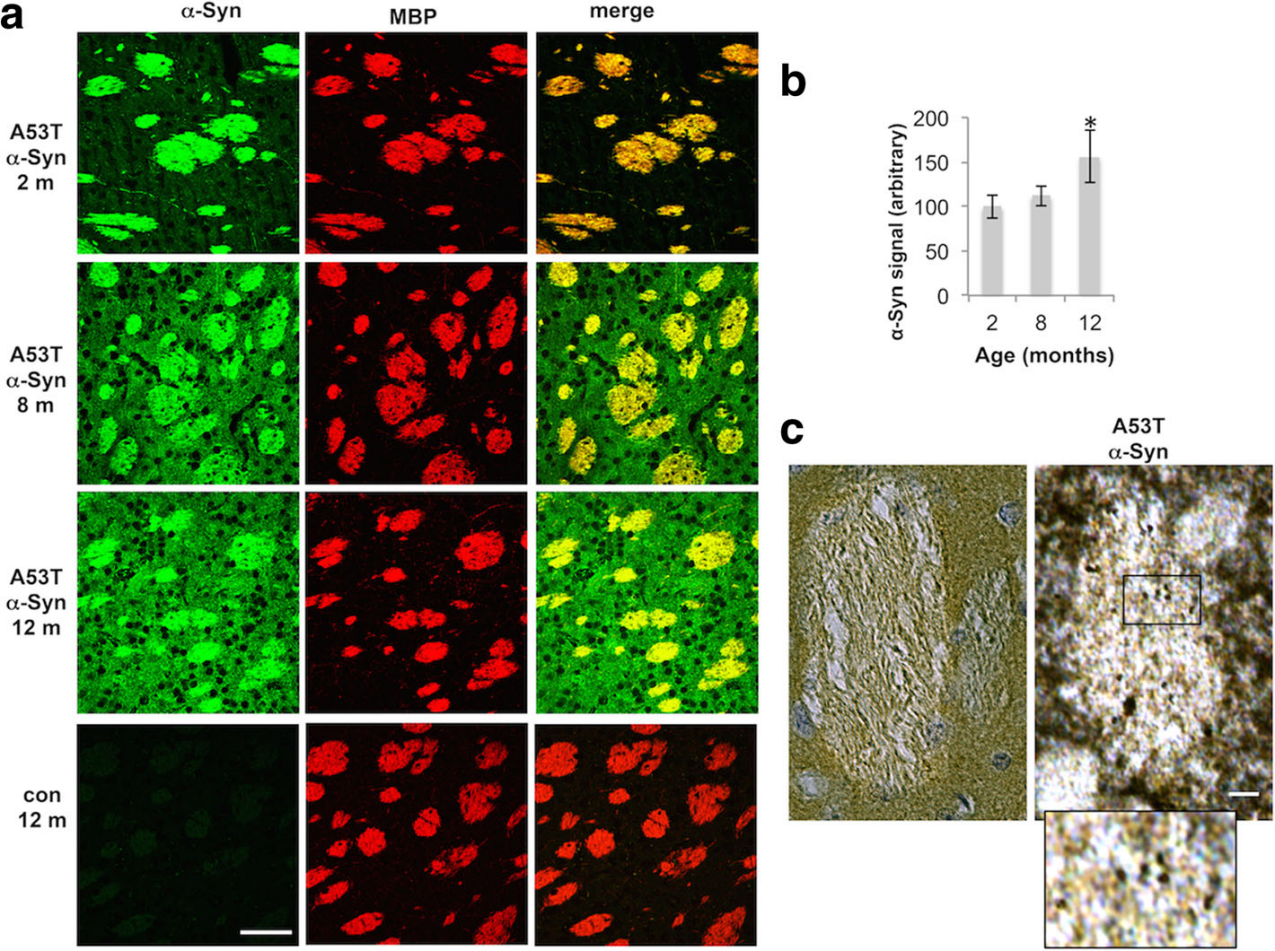
Age-dependent accumulation of α-Syn toxicity in the striatum. **a** Coronal brain sections containing rostral-dorsal striatum (6 μm, paraffin embedded) of A53T α-Syn mouse at 2, 8 and 12 months of age, or control brain section at 12 months old, immunoreacted with anti-α-Syn antibody (syn - 303). Scale bar, 50 μm **b**. Quantification of α-Syn signal in striatum of *n* = 4 mice at each age group. Mean ± SD. *, < 0.05, one-way ANOVA **c**. Coronal brain sections of A53T α-Syn and control mouse brains, at 12 months of age. Immunoreacted with anti-α-Syn antibody followed by Avidin/Biotin detection. Scale bar, 10 μm. Square present area of magnification

To find out whether the age-dependent, localized loss of MBP signal is associated with the occurrence of α-Syn toxicity, we stained consecutive brain sections of A53T α-Syn mice at 2, 8 and 12 months for α-Syn and MBP. By IHC, the signal for transgenic α-Syn overexpression could not be distinguished from that of pathogenic α-Syn, yet increases in α-Syn levels, which are strongly associated with α-Syn toxicity, were detected in an age-dependent manner. Interestingly, the distribution of α-Syn signal in matrix and striosomes was also affected in an age-dependent manner. At 2 months of age, α-Syn signal was mostly in striosomes and a very low signal could be detected in the matrix. However, at 8 months of age, α-Syn signal was detected both in matrix and striosomes and at 12 months of age, the degree of α-Syn signal in matrix was even higher (Fig. 7a). Quantifying α-Syn immunoreactivity in the striatum, including matrix and striosomes, we detected a significantly higher signal in 12 month-old A53T α-Syn brains (Fig. 7b). That is, relative to control mice, A53T α-Syn mice had an α-Syn signal of 156.5 ± 18.7% (mean ± SD, *n* = 4 *p* < 0.05, one-way ANOVA).

Finally, we searched for evidence of α-Syn pathology in striosomes and matrix of A53T α-Syn tg mouse brains using anti-α-Syn antibody. A punctuated α-Syn immunoreactive signal was abundantly detected in striosomes of 12–14 months-old A53T α-Syn tg mice (Fig. 7c). This signal was not detected in striosomes of the 2 or 8 months-old brains. α-Syn signal in the matrix of 12–14 month-old mouse brains appeared diffused with no obvious differences from the 8 months-old mice. We therefore concluded that the age-dependent MBP loss, localized to striosomes, is associated with the occurrence of α-Syn pathology.

## Discussion

We report herein higher levels of phospholipids in myelin purified from either PrP-A53T α-Syn or Thy-1 human wt α-Syn mouse brains, than in the respective control brains. The increases in phospholipid levels were detected at the age of 4–6 months, representing fully developed and apparently healthy mice. Analysis of myelin ultrastructure in the PrP-A53T α-Syn mouse brains indicated intact myelin at this age. A systematic analysis revealed no myelin loss before 8 months of age. In addition, we detected evidence for an inhibitory effect of α-Syn on membranous sheets formation *in vitro*, in cultured oligodendrocytes. Together, the results herein show a previously unreported effect of neuronal α-Syn on myelin phospholipid composition and suggest that axonal hypomyelination is associated with the occurrence of α-Syn toxicity rather than with altered phospholipid content.

In this study we utilized two mouse models for PD, in which α-Syn is expressed in neurons. These mouse models recapitulate behavioral and pathological abnormalities of PD and to a certain degree also DLB. Whereas the PrP promoter mediates primarily neuronal expression, it can also regulate non-neuronal cell expression [12]. In contrast, the Thy-1 promoter is considered a neuron-specific promoter [28]. It is now accepted that under pathogenic conditions, neuronal α-Syn is secreted and can accumulate in other neural or glial cells. This mechanism was shown to be associated with the spread of α-Syn toxicity and was referred to as a prion-like property of α-Syn. Neural secretion of α-Syn is enhanced with accumulation of pathogenic α-Syn species and by various stress conditions [23]. Based on this property of α-Syn, it is difficult to determine the mechanism through which α-Syn acts to control myelin phospholipid content. Specifically, are the cellular signals resulting in higher levels of myelin phospholipids primary axonal or primary oligodendroglial signals? We show that the effect of α-Syn on phospholipid levels occurs in the absence of evidence for neuronal or oligodendroglial pathologies. Based on the results, we suggest that regulation of myelin phospholipid levels may be a physiological activity of α-Syn. However, we cannot rule out that a low amount of neuronally secreted or pathogenic α-Syn could potentially underlie these findings.

Myelin instability and loss is regarded as an early event in the pathogenesis of the synucleinopathy MSA. A recent study showed that myelin lipids, e.g., sphingomyelin, sulfatide and galactosylceramide, were severely decreased in white matter collected from disease-affected regions of MSA brains [2, 15]. Mouse models for MSA, in which transgenic α-Syn is expressed in oligodendroglial cells, recapitulated MSA pathogenesis to a high degree [1]. That is, α-Syn expression under the control either of PLP, MBP or CNPase oligodendroglial promoters, resulted in its accumulation in cytoplasmic inclusions in oligodendrocytes and motor deficits. However, the effect of oligodendrocytic α-Syn expression on myelin abnormalities varied between the models. No obvious oligodendroglial or myelin loss were detected in PLP-α-Syn mice [31]; however, myelin abnormalities and loss were prominent in the MBP-α-Syn and CNPase-α-Syn mice [58, 71]. The CNPase-α-Syn mice have demonstrated a primary oligodendroglial disease, where oligodendrocytic degeneration causes demyelination and a secondary axonal degeneration [71]. Importantly, the findings of those studies differed from those of the present study, which examined PD models in which α-Syn is expressed in neurons. Specifically, in the PD models, increases in phospholipid content were detected; alterations in lipid content were associated with healthy mice, whereas evidence of myelin loss was associated with accumulation of α-Syn pathology; no associated loss of myelin proteins was detected in the young mice; no evidence for oligodendroglial pathology in the form of GCI or accumulation of α-Syn were detected. Together, this comparison supports our argument that α-Syn’s effect on phospholipids is a result of physiological, neuronal α-Syn expression rather than a pathogenic effect of this protein.

We utilized P25α as a marker for myelinating oligodendrocytes. We counted the number of oligodendrocytes in the striatum and corpus callosum and found no difference between A53T α-Syn and control mice. However, the intensity of the P25α signal was lower in the old, symptomatic A53T α-Syn brains than in control mouse brains. In addition, lower P25α levels were detected by Western blotting in samples of 10 month-old Thy-1 α-Syn than in control mouse brains. P25α expression is critical for the differentiation of oligodendrocytes and its levels increase with maturation of oligodendrocytes [24, 35]. In multiple sclerosis, lower P25α levels were associated with a lower remyelinating activity at relapse stage [27]. The lower P25α signal we detected in A53T α-Syn tg brains may therefore suggest affected myelination in older α-Syn mouse brains. Importantly, we found no evidence for abnormal accumulation of P25α in neuronal or glial cytoplasmic inclusions [35], supporting our conclusion for a mechanism of toxicity that is independent of MSA.

The results obtained *in vitro* with primary oligodendrocytes suggest that α-Syn inhibits maturation and differentiation of oligodendrocytes. Hence, oligodendrocyte precursor cells, which might be recruited and replace dysfunctional oligodendrocytes, are compromised. This effect of α-Syn may result from neuronally secreted α-Syn that is taken up by oligodendrocytes, as we have shown previously [33], and contribute to pathological consequences on myelination in PD. Of note, it is not clear whether or to what degree α-Syn toxicity is enhanced by axonal hypomyelination. Interestingly, a potential association between hypomyelination and α-Syn pathology was recently suggested by Braak and co-authors, who reported that α-Syn pathology is more evident in un-myelinated or thinly myelinated axons [10]. It is still unclear which is the result and which the consequence: Does axonal hypomyelination enhance α-Syn pathology? or *vice verse*, Does α-Syn pathology enhance hypomyelination of axons?

A characteristic biochemical feature of myelin that distinguishes it from most biological membranes is its high lipid-to-protein ratio: lipids account for at least 70% of its dry weight. The most abundant lipid groups in myelin are cholesterol, phospholipids and glycosphingolipids. Phospholipids represent about 40% of total lipids in myelin membrane [13, 49, 56]. This is lower then their relative amount in most membranes, which is ~65% [13, 49, 56]. The most abundant phospholipid in myelin is ethanolamine plasmalogen. Its exceptionally high levels in myelin membrane are a characteristic feature; however, its role in myelin structure or function is poorly understood. In humans, the total amount of brain plasmalogens increases dramatically during the developmental phase of myelination and reaches maximum levels by around the age of 30 years [41]. Later on, plasmalogen content generally decreases with age [19, 37]. The importance of plasmalogens is emphasized by the consequences of defects in plasmalogen biosynthesis, which in humans cause the fatal disease rhizomelic chondrodysplasia punctata (RCDP; [63]). Decreases in ethanolamine plasmalogen levels are associated with human diseases, such as Alzheimer’s disease [11]. We detected higher levels of ethanolamine plasmalogen in myelin from healthy A53T α-Syn and Thy-1 α-Syn tg mouse brains. To the best of our knowledge, higher ethanolamine plasmalogen levels are not associated with neurodegeneration. It is possible that the unique structure of the ether based plasmalogen decreases the fluidity and increases the hydrophobicity of myelin. Therefore, the higher levels of ethanolamine plasmalogen we detected may further increase the myelin packing density [56] as part of myelin formation.

## Conclusions

We performed a systematic study to understand the effect of neuronal-expressed α-Syn on myelin composition. We found that α-Syn expression increased the levels of phospholipids in the absence of evidences for the occurrence of α-Syn or related-pathologies. We concluded that α-Syn effect on myelin composition is an early event in the sequence of events leading to axonal loss and neurodegeneration.
